# JOINT TRAJECTORIES OF FRAILTY AND COGNITIVE FUNCTION IN OLDER KOREANS: A 14-YEAR ANALYSIS WITH NATIONAL-LEVEL DATA

**DOI:** 10.1093/geroni/igae098.2766

**Published:** 2024-12-31

**Authors:** Yunkyung Jung, Jiyoung Lyu

**Affiliations:** Hannam University, Daejeon, Republic of Korea; Hallym University, Chuncheon, Kangwon-do, Republic of Korea

## Abstract

Despite the evidence on the link between frailty and cognitive function, less is known about the joint developments of these two multidimensional indexes. The present study aimed to identify distinct trajectories of frailty and cognitive function and examine the influence of sociodemographic and lifestyle factors on the co-development of frailty and cognitive function in older Koreans. A nationally representative sample of 2,766 adults aged ≥65 years from the first (2006) to the eighth (2020) waves of the Korean Longitudinal Study of Aging (KLoSA) was analyzed. Frailty was measured with a Frailty Index with 33 deficit-related indicators. Cognitive function was measured using the Mini-Mental State Examination (MMSE). Group-based trajectory modeling was used to fit the developments of frailty and cognitive function. A multinomial logistic regression was conducted to assess the predictors for each trajectory group. Three joint trajectories were identified, including cognitive frailty (13.2%), cognitive decline and progressive frailty (33.0%), and no cognitive frailty (53.7%). Compared to individuals in the no cognitive frailty group, those in the cognitive frailty group were more likely to be unemployed and current smokers; while those in the cognitive decline and progressive frailty group were more likely to be women, have low income, and not current drinkers. Significant heterogeneity in the co-development of frailty and cognitive function in older Koreans was identified. Findings suggest that different sociodemographic and lifestyle factors determined the trajectory membership.

